# Tolerance to bronchodilation during treatment with long-acting beta-agonists, a randomised controlled trial

**DOI:** 10.1186/1465-9921-6-107

**Published:** 2005-09-16

**Authors:** Sarah Haney, Robert J Hancox

**Affiliations:** 1 Freeman Hospital, Newcastle-upon-Tyne, UK; 2 Department of Respiratory Medicine, Waikato Hospital, Hamilton, New Zealand

## Abstract

**Background:**

Regular use of beta-agonists leads to tolerance to their bronchodilator effects. This can be demonstrated by measuring the response to beta-agonist following bronchoconstriction using methacholine. However most studies have demonstrated tolerance after a period of beta-agonist withdrawal, which is not typical of their use in clinical practice. This study assessed tolerance to the bronchodilator action of salbutamol during ongoing treatment with long-acting beta-agonist.

**Methods:**

Random-order, double-blind, placebo-controlled, crossover trial. After 1 week without beta-agonists, 13 asthmatic subjects inhaled formoterol 12 μg twice daily or matching placebo for 1 week. Eight hours after the first and last doses subjects inhaled methacholine to produce a 20% fall in FEV_1_. Salbutamol 100, 200 and 400 μg (cumulative dose) was then given at 5-minute intervals and FEV_1 _was measured 5 minutes after each dose. After a 1 week washout subjects crossed over to the other treatment. Unscheduled use of beta-agonists was not allowed during the study. The main outcome variable was the area under the salbutamol response curve.

**Results:**

The analysis showed a significant time by treatment interaction indicating that the response to salbutamol fell during formoterol therapy compared to placebo. After 1 week of formoterol the area under the salbutamol response curve was 48% (95% confidence interval 28 to 68%) lower than placebo. This reduction in response remained significant when the analyses were adjusted for changes in the pre-challenge FEV_1 _and dose of methacholine given (p = 0.001).

**Conclusion:**

The bronchodilator response to salbutamol is significantly reduced in patients taking formoterol. Clinically relevant tolerance to rescue beta-agonist treatment is likely to occur in patients treated with long-acting beta-agonists.

## Background

Long-acting beta-agonists are often added to inhaled corticosteroids to improve asthma control.[1] Despite this, most patients still need a short-acting beta-agonist for relief of breakthrough symptoms. The possibility that chronic long-acting beta-agonist use might adversely affect the acute response to short-acting beta-agonists is rarely considered.

It is well known that regular use of long-acting beta-agonists leads to tolerance to their bronchoprotective effects (their ability to prevent bronchoconstriction).[2,3] Studies looking for bronchodilator tolerance have had more variable results.[4,5] This has led to a widespread belief that clinically significant tolerance to bronchodilation does not occur.[6] However, several recent studies have clearly shown that bronchodilation tolerance does occur and becomes more apparent with increasing levels of bronchoconstriction. [7-11] This raises the possibility that beta-agonists will be less effective during acute severe asthma in patients using long-acting beta-agonists.

Much of the evidence for bronchodilator tolerance has come from studies using a 'challenge-rescue model' that measures the response to short-acting beta-agonists after bronchoconstriction has been induced with either methacholine or exercise.[7-11] Testing bronchodilation from a state of increased bronchomotor tone is thought to mimic patients' use of beta-agonists to relieve asthma symptoms. However, many of these studies have been performed after a period of withdrawal from maintenance beta-agonist [7,8,11] and the findings may not be relevant to patients who continue to use their long-acting beta-agonist twice daily. Other studies have been carried out at the time of peak effect of long-acting beta-agonist, when subjects are least likely to need additional bronchodilator and when results are confounded by differing baseline levels of bronchodilation and bronchial reactivity.[9,10]

The bronchodilator and bronchoprotective effects of long-acting beta-agonists peak within 1 hour of inhalation.[12] If they are taken twice daily as recommended then patients will be most vulnerable to bronchoconstriction and most likely to need their reliever inhalers 8–12 hours after inhalation. Tolerance to the bronchodilator effects of these inhalers at this time is therefore of the highest clinical relevance. Although tolerance to bronchoprotection is known to occur at this time,[3] changes in bronchodilation using this 'challenge-rescue'[10] model have not been studied in placebo-controlled trials.

This study was designed to assess tolerance to salbutamol bronchodilation 8 hours after regular formoterol treatment in a double-blind, randomised, placebo-controlled, cross-over trial.

## Methods

Subjects had a physician diagnosis of asthma and a PD_20 _methacholine (provocative dose of methacholine required to produce a 20% fall in FEV_1_) <1.5 mg (<7.7 μmol). Those currently using long-acting beta-agonists were excluded. Those who had used oral corticosteroids in the previous 3 months or who had changed asthma treatment in the previous 6 weeks were excluded, as were pregnant and lactating women. All subjects provided written informed consent. Ethical approval for the study was granted by the Waikato Ethics Committee.

After a 1-week run-in period during which no beta-agonists were used, subjects were computer randomised to receive coded bottles of capsules containing formoterol 12 μg or matching placebo for use with the Foradil aerolizer device (Foradil, Novartis, Auckland, New Zealand). A methacholine challenge and salbutamol response was performed 8 hours after the first dose of study medication. The study medication was then taken twice daily for 1 week. A further challenge and response was performed 8 hours following the last dose. After a washout period of at least 1 week the study was repeated using the alternative medication. Use of additional beta-agonists was not allowed throughout the study. Ipratropium bromide (20 μg Atrovent metered dose inhaler, Boehringer Ingelheim, Auckland, New Zealand) was used for symptom relief.

Methacholine challenge was performed using a modified Yan technique.[13] Baseline FEV_1 _was the highest of 3 consistent measurements. Subjects then inhaled doubling doses of nebulised methacholine from 0.0073 mg to 3.728 mg from a dosimeter. FEV_1 _was measured 1 minute after each dose. Once the FEV_1 _had fallen by ≥20% from baseline, methacholine challenge was stopped. The PD_20 _(cumulative dose) was calculated by linear interpolation. Where the FEV_1 _fell <20% from baseline (2 subjects each on 1 occasion after formoterol) an arbitrary PD_20 _of 15 mg was assigned (twice the maximum cumulative dose given).

Salbutamol (Ventolin, GlaxoSmithKline, Auckland, New Zealand) 100 μg, 100 μg and 200 μg via metered dose inhaler and Volumatic spacer was given at 0, 5 and 10 minutes after challenge respectively. The FEV_1 _was measured 5 minutes after each dose of salbutamol, giving a total response time of 15 minutes.

The main outcome measurement was the area under the salbutamol response curve (AUC), expressed as a percentage of the methacholine-induced fall in FEV_1_. A secondary outcome was the final (15-minute) FEV_1 _after 400 μg salbutamol. Previous studies indicate that the pre-methacholine FEV_1 _and dose of methacholine used are significant covariates of the post-methacholine bronchodilator response [7] and analyses adjusted for these covariates using analysis of covariance. Methacholine doses were log-transformed for analysis to approximate a normal distribution. The test of the hypothesis used a general linear model with terms for subject, drug and time as well as the covariates of pre-methacholine FEV_1 _and log methacholine dose. Whether tolerance to bronchodilation occurred was assessed using the drug*time interaction term in the model. Post-hoc comparisons of the AUC and final FEV_1 _between placebo and formoterol treatments on day 1 and after 1 week of treatment used Tukey's exact method. Comparisons of pre-challenge FEV_1 _and PD_20 _methacholine use paired t tests. Analyses were performed using *Minitab 13.2 *and *SPSS 10.0 *for *Windows*. All available data were used in the main analysis. One subject did not attend for the second placebo challenge. Excluding this subject did not significantly alter the analysis.

## Results

Eighteen subjects started the run-in period. Five subjects withdrew – one for personal reasons, one because he was prescribed oral steroids for acute gout, two for respiratory tract infections and one for an exacerbation of asthma. Thirteen subjects (5 male) completed the study. One subject did not attend for the challenge after 1 week of placebo therapy. Baseline data on the subjects are presented in table 1.

**Table 1:** Baseline data on subjects.

Subject	Age (years)	Sex	Dose of inhaled steroids (mcg)*	FEV_1 _at 1^st ^placebo challenge (L)	Percent predicted FEV_1_
1	34	Female	200	3.11	100
2	40	Female	400	2.60	89
3	26	Male	1000	3.52	85
4	23	Male	0	4.32	97
5	31	Female	0	2.41	86
6	36	Male	0	4.44	107
7	31	Female	0	3.09	98
8	19	Female	0	3.83	110
9	25	Male	0	3.93	87
10	23	Female	250	2.43	73
11	60	Female	400	1.73	73
12	24	Female	0	2.87	89
13	24	Male	500	4.18	90

* budesonide equivalent (beclometasone = budesonide = 2 × fluticasone)

### Changes in baseline FEV_1 _and PD_20_

The pre-challenge FEV_1 _was higher during formoterol treatment than during placebo. This was of borderline significance after 1 week (table 2). The first dose of formoterol also increased the PD_20 _methacholine. After 1 week of formoterol this protection had decreased and the PD_20 _was not significantly different to placebo (table 2).

**Table 2:** Changes in FEV_1_, PD_20 _and fall in FEV_1_

		**Day 1**	**Day 8**
**Pre-methacholine FEV_1_(L) **Mean and SD	Placebo	3.26 (0.84)	3.24 (0.86)
	Formoterol	3.44 (0.80)	3.36 (0.89)
	Difference (95% CI)	0.18 (0.08, 0.28)	0.12 (-0.01. 0.24)
		P = 0.002	P = 0.069

**PD_20_(mg) **Geometric mean and 95% CI for mean	Placebo	0.10 (0.04, 0.24)	0.12 (0.04, 0.32)
	Formoterol	0.45 (0.15, 1.35)	0.17 (0.05, 0.60)
	Difference (doubling doses) (95% CI)	2.12 (0.88, 3.36)	0.51 (-0.49, 1.51)
		P = 0.003	P = 0.286

**Fall in FEV_1 _from baseline (%) **Mean and SD	Placebo	24.78 (4.74)	23.73 (4.54)
	Formoterol	24.56 (5.24)	22.26 (4.34)
	Difference (95% CI)	-0.22 (-3.54, 3.10)	-1.47 (-4.61, 1.66)
		P = 0.886	P = 0.327

N = 13. As there is no data on subject 6 for day 8 placebo, data from day 1 have been used to calculate means.

### Changes in salbutamol response

The area under the salbutamol response curve was lower during formoterol therapy compared to placebo. There was a significant time*treatment interaction indicating that the change in salbutamol response from day 1 to day 8 of formoterol treatment was different to the change in response from day 1 to day 8 of placebo (table 3). Comparisons between placebo and formoterol at each time point found that the reduction in AUC was not statistically significant after the first dose of formoterol, after adjusting the analysis for the increased dose of methacholine used. However, after 1 week of treatment the reduction was statistically significant even after adjusting for the dose of methacholine and the pre-challenge FEV_1 _(table 4). The mean difference in adjusted AUC between the day 1 and day 8 formoterol challenges was 58.1%.time (95% confidence interval 2.7, 113.4; p = 0.04).

**Table 3:** ANOVA table for AUC.

Source	Degrees of freedom	Seq sums of squares	Adjusted mean squares	F	P
Baseline FEV1	1	2195	0	0	0.998
Log dose methacholine	1	88136	46295	22.13	0.000
Subject	12	83869	5368	2.57	0.016
Time	1	10109	5292	2.53	0.121
Treatment	1	24752	18182	8.69	0.006
Time*treatment	1	14283	14283	6.83	0.013
Error	33				
Total	50				

**Table 4:** Differences in area under the salbutamol response curve (AUC)

		**Day 1**	**Day 8**
**AUC as % of fall in FEV1 (%.time) **mean and SD	Placebo	196 (74)	205 (63)
	Formoterol	120 (61)	107 (53)
	Difference (least squares means, 95% confidence interval)	13.06 (-50.4, 76.53)	85.44 (31.78, 139.1)
		P = 0.944	P = 0.001

**Final FEV_1_(L) **mean and SD	Placebo	3.32 (0.84)	3.31 (0.89)
	Formoterol	3.25 (0.22)	3.12 (0.84)
	Difference (least squares means, 95% confidence interval)	0.06 (-0.18, 0.30)	0.26 (0.06, 0.46)
		P = 0.909	P = 0.006

Differences in AUC by treatment and time using Tukey's exact method from covariate analysis (n = 13)

**Figure 1:**
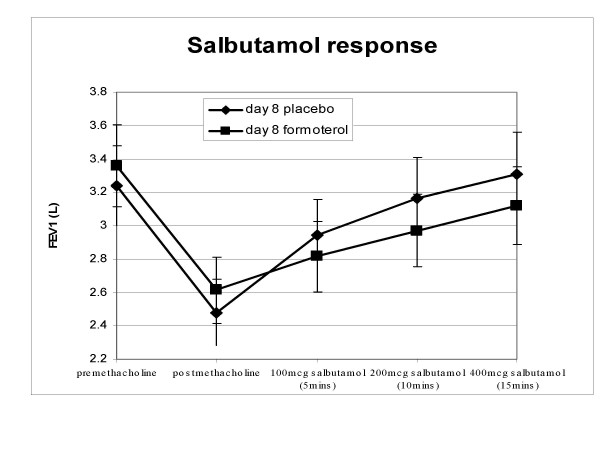
Response to salbutamol following methacholine challenge. Mean and standard error.

Despite the fact that pre-challenge FEV_1 _and post-methacholine FEV_1 _were higher during formoterol treatment, the FEV_1 _at the end of the salbutamol dose-response was lower on both formoterol days compared to placebo (see figure 1 for day 8). This difference was not statistically significant after 1 dose, but after 1 week of formoterol the mean difference was significant after allowing for covariates (table 4).

There was no effect of order of treatment on salbutamol response, PD_20 _or FEV_1_. Analysing AUC as absolute change in FEV_1 _instead of as a percentage of the fall in FEV_1 _gave similar results. In this study, baseline FEV_1_ was not a significant covariate in the analysis of AUC. Excluding this from the model did not materially alter the results of the analysis. There was no difference in the change in salbutamol response between those subjects taking and those not taking inhaled corticosteroids.

## Discussion

This study has demonstrated a marked reduction in the bronchodilator response to salbutamol within the usual dosing interval of standard doses of formoterol (12 μg bd). After 1 week of formoterol therapy the area under the salbutamol response curve in the formoterol arm was nearly half that of placebo. This reduction in response was statistically significant after adjusting for the pre-challenge FEV_1 _and dose of methacholine used in the challenge

The area under the salbutamol response curve was also reduced after a single dose of formoterol. However, this was not statistically significant after adjusting for the dose of methacholine used in the challenge, which was higher after the first dose of formoterol. After 1 week of formoterol therapy the PD_20 _methacholine was not significantly different to placebo, indicating that tolerance to bronchoprotection had occurred. Hence at this time point there was little protection against bronchoconstriction and greatly reduced bronchodilation.

It is known that cellular tolerance to beta-agonists occurs very rapidly, within 8 hours *in vitro*, [14] so it is possible that 'tolerance' had occurred even prior to the challenge on day 1 of formoterol treatment. However, there may be other explanations for a reduced response to salbutamol during formoterol therapy, including the fact that beta_2_-receptors continue to be occupied by formoterol 8 hours after the last dose, leaving fewer receptors available to bind salbutamol. This could explain the reduction in the salbutamol response after the first dose of formoterol. However, the analysis showed a significant interaction between time and treatment, indicating that the bronchodilator response to salbutamol declined further during the formoterol treatment period. Lee et al [15] also found that the salbutamol response 1 hour after a single dose of salmeterol or formoterol was significantly lower after 1 week of treatment than after the first dose of long-acting beta-agonist. If receptor occupancy was the only explanation for the reduction in AUC we would expect to have found a similar reduction in AUC on the first and last day of formoterol treatment. Moreover, the reduction in salbutamol response after a week of formoterol treatment observed in this study was similar to a previous study in our laboratory, where the salbutamol response was measured 24 hours after the last dose of formoterol.[16] Receptor occupancy by formoterol would be much lower at this time, suggesting that the reduction in response is more likely to be due to receptor downregulation.

Regardless of the mechanism, we have shown that the bronchodilator response to salbutamol is reduced during long-acting beta-agonist treatment. Previous studies have been criticised for only analysing changes in FEV_1 _from baseline rather than actual post-bronchodilator levels of FEV_1_.[17] It is notable in this study that the final FEV_1 _after 400 μg of salbutamol was lower during formoterol treatment than during placebo, despite a higher pre-methacholine FEV_1 _(figure 1).

A recent meta-analysis of studies of regular beta-agonist therapy concluded that the bronchodilator response to subsequent beta-agonist is reduced.[18] However, most of the individual studies included in this analysis failed to show significant tolerance. In contrast, all of the published trials that have measured the bronchodilator response after methacholine challenge have shown significant tolerance.[7-9,11] The 'challenge-rescue model' appears to be more sensitive to changes in the response to bronchodilators. Bronchodilation is a 'closed-end scale'[19] with a maximal achievable level. The dose-response to beta-agonist bronchodilators is therefore dependent on the pre-bronchodilator FEV_1_.[20] Subjects with mild, stable asthma are often close to their maximum FEV_1 _and have little capacity for additional bronchodilation. Using methacholine to induce a controlled level of bronchoconstriction before testing bronchodilators ensures that there is capacity for bronchodilation and differences in responses can be shown more easily. Since asthmatics take bronchodilators to relieve symptoms caused by bronchoconstriction it seems logical to test bronchodilators from a state of bronchoconstriction.

This study is the first blinded, placebo-controlled trial to test for bronchodilator tolerance during the trough period of long-acting beta-agonist dosing using the 'challenge-rescue' model. A recent non-blinded trial compared the bronchodilator response to salbutamol after methacholine challenge between formoterol/budesonide or salmeterol/fluticasone to inhaled steroids alone, at 12 hours after inhalation.[21] This study found a similar degree of tolerance to the present study despite methodological differences including the use of salbutamol as-needed.

International guidelines recommend that long-acting beta-agonists are used only in those subjects already taking inhaled corticosteroids.[1] Not all of our subjects were taking such medication. However, this does not detract from the applicability of our study as it has been established that stable doses of inhaled corticosteroids do not alter the development of tolerance to beta-agonists.[7] Moreover, it is widely recognised that patients are often poorly compliant with their inhalers and it is likely that many patients will continue to use their long-acting beta-agonists without inhaled corticosteroids.

**Figure 2:**
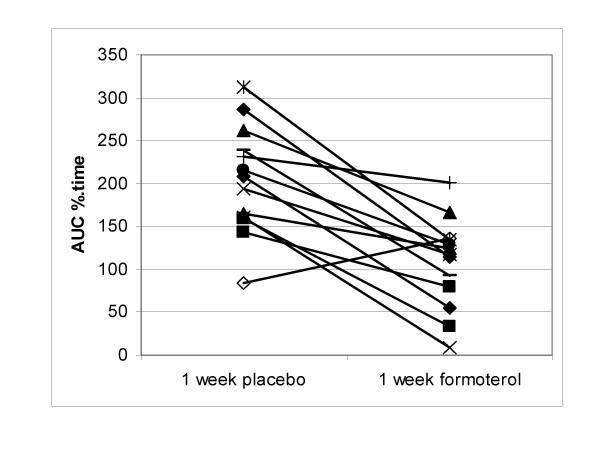
**Individual data for AUC after 1 week of placebo and formoterol**. AUC is expressed as a percentage of the fall in FEV_1 _induced by methacholine.

All but one of our subjects showed some decrease in the bronchodilator ability of salbutamol after 1 week of formoterol treatment, although the magnitude of this was variable (figure 2). The reasons for this variability are unknown. Lee et al found that tolerance occurred to the same extent in subjects who were homozygous for the Arg-16 or for the Gly-16 polymorphisms of the beta_2_-receptor.[15]

## Conclusion

In conclusion, we have found that bronchodilator tolerance is present during the usual dosing interval of long-acting beta-agonists. Wraight found that tolerance to salbutamol bronchodilation increased with increasing levels of bronchoconstriction.[11] The level of bronchoconstriction induced in this study (20%) was mild compared to that likely to occur during a severe exacerbation of asthma. Patients using long-acting beta-agonists who experience exacerbations of asthma may have an inadequate response to beta-agonist relievers.

## Competing interests

The author(s) declare that they have no competing interests.

## Authors' contributions

RH conceived the trial, participated in its design, interpretation of results and helped to draft the manuscript. SH participated in the design of the study, acquired the data, performed the statistical analysis and helped to draft the manuscript. Both authors read and approved the final manuscript.
